# A Recessive Founder Mutation in Regulator of Telomere Elongation Helicase 1, *RTEL1*, Underlies Severe Immunodeficiency and Features of Hoyeraal Hreidarsson Syndrome

**DOI:** 10.1371/journal.pgen.1003695

**Published:** 2013-08-29

**Authors:** Bari J. Ballew, Vijai Joseph, Saurav De, Grzegorz Sarek, Jean-Baptiste Vannier, Travis Stracker, Kasmintan A. Schrader, Trudy N. Small, Richard O'Reilly, Chris Manschreck, Megan M. Harlan Fleischut, Liying Zhang, John Sullivan, Kelly Stratton, Meredith Yeager, Kevin Jacobs, Neelam Giri, Blanche P. Alter, Joseph Boland, Laurie Burdett, Kenneth Offit, Simon J. Boulton, Sharon A. Savage, John H. J. Petrini

**Affiliations:** 1 Clinical Genetics Branch, Division of Cancer Epidemiology and Genetics, National Cancer Institute, Rockville, Maryland, United States of America; 2 Clinical Genetics Service, Department of Medicine, Memorial Hospital, Memorial Sloan-Kettering Cancer Center, New York, New York, United States of America; 3 Molecular Biology Program, Sloan Kettering Institute, Memorial Sloan-Kettering Cancer Center, New York, New York, United States of America; 4 DNA Damage Response Laboratory, London Research Institute, Cancer Research UK, South Mimms, United Kingdom; 5 Department of Pediatrics, Memorial Sloan-Kettering Cancer Center, New York, New York, United States of America; 6 Department of Pathology, Memorial Sloan-Kettering Cancer Center, New York, New York, United States of America; 7 Cancer Genomics Research Laboratory, SAIC-Frederick, Inc., NCI-Frederick, Frederick, Maryland, United States of America; 8 Cancer Genetics and Biology Program, Sloan-Kettering Institute, Memorial Sloan-Kettering Cancer Center, New York, New York, United States of America; Centre for Cancer Biology, SA Pathology, Australia

## Abstract

Dyskeratosis congenita (DC) is a heterogeneous inherited bone marrow failure and cancer predisposition syndrome in which germline mutations in telomere biology genes account for approximately one-half of known families. Hoyeraal Hreidarsson syndrome (HH) is a clinically severe variant of DC in which patients also have cerebellar hypoplasia and may present with severe immunodeficiency and enteropathy. We discovered a germline autosomal recessive mutation in *RTEL1*, a helicase with critical telomeric functions, in two unrelated families of Ashkenazi Jewish (AJ) ancestry. The affected individuals in these families are homozygous for the same mutation, R1264H, which affects three isoforms of *RTEL1*. Each parent was a heterozygous carrier of one mutant allele. Patient-derived cell lines revealed evidence of telomere dysfunction, including significantly decreased telomere length, telomere length heterogeneity, and the presence of extra-chromosomal circular telomeric DNA. In addition, *RTEL1* mutant cells exhibited enhanced sensitivity to the interstrand cross-linking agent mitomycin C. The molecular data and the patterns of inheritance are consistent with a hypomorphic mutation in *RTEL1* as the underlying basis of the clinical and cellular phenotypes. This study further implicates *RTEL1* in the etiology of DC/HH and immunodeficiency, and identifies the first known homozygous autosomal recessive disease-associated mutation in *RTEL1*.

## Author Summary

Patients with dyskeratosis congenita (DC), a rare inherited disease, are at very high risk of developing cancer and bone marrow failure. The clinical features of DC include nail abnormalities, skin discoloration, and white spots in the mouth. Patients with Hoyeraal-Hreidarsson syndrome (HH) have symptoms of DC plus cerebellar hypoplasia, immunodeficiency, and poor prenatal growth. DC and HH are caused by defects in telomere biology; improperly maintained telomeres are thought to be a major contributor to carcinogenesis. In half the cases of DC, the causative mutation is unknown. By studying families affected by DC for whom a causative mutation has not yet been identified, we have discovered a homozygous germline mutation in *RTEL1*, a telomere maintenance gene that, if mutated, can result in HH. The mutations result in the inability of the RTEL1 protein to function properly at the telomere, and underscore its important role in telomere biology.

## Introduction

Hoyeraal Hreidarsson syndrome (HH) is a clinically severe variant of the telomere biology disorder dyskeratosis congenita (DC) [1]. DC is a heterogeneous inherited bone marrow failure syndrome (IBMFS) diagnosed by the presence of the classic triad of dysplastic nails, abnormal skin pigmentation, and oral leukoplakia. However, substantial clinical heterogeneity has been observed and the phenotype may include pulmonary fibrosis, liver disease, esophageal, urethral, or lacrimal duct stenosis, developmental delay, and/or other complications. Individuals with DC are at very high risk of bone marrow failure (BMF), myelodysplastic syndrome, and cancer [2]. The clinical consequences of DC manifest at variable ages and in different patterns, even within the same family. Independent of the classic triad, lymphocyte telomere lengths less than the first percentile for age are diagnostic of DC [3]. Depending on the affected gene, DC can be inherited in X-linked recessive (XLR), autosomal dominant (AD), or autosomal recessive (AR) patterns. Germline mutations in *DKC1* result in XLR inheritance, mutations in *TERC*, *TERT*, *RTEL1*, or *TINF2* result in AD inheritance, and mutations in *TERT*, *RTEL1*, *CTC1*, *NOP10*, *NHP2*, or *WRAP53* result in AR inheritance [4]–[7] [8]; mutations in these genes account for approximately one-half of classic DC cases.

Patients with HH have many of the DC features listed above; however, severe immunodeficiency [9], non-specific enteropathy, intrauterine growth retardation (IUGR), and developmental delay may be the presenting features. In addition to features of DC, the presence of cerebellar hypoplasia is often the basis for a diagnosis of HH [1]. Patients with HH have extremely short telomeres, even when compared with other DC patients [3]. Germline mutations in *DKC1* (XLR), *TINF2* (AD), or *TERT* (AR) have been shown to cause HH. The causative mutation in HH is known in less than one-half of cases.

We clinically characterized individuals with HH from two different families. The affected individuals had IUGR, immunodeficiency, enteropathy, and extremely short telomeres. In both families, we discovered homozygous recessive germline mutations in Regulator of Telomere Elongation Helicase 1 (*RTEL1*) and characterized the telomere defect that resulted from these mutations. While *RTEL1* mutations have been previously implicated in AD and AR compound heterozygous cases of DC, HH, and DC-like cases [6], [7], this report is the first instance of a homozygous DC-causative mutation in this gene.

## Results

### Clinical Characterization

#### Family NCI-318

**Figure 1:**
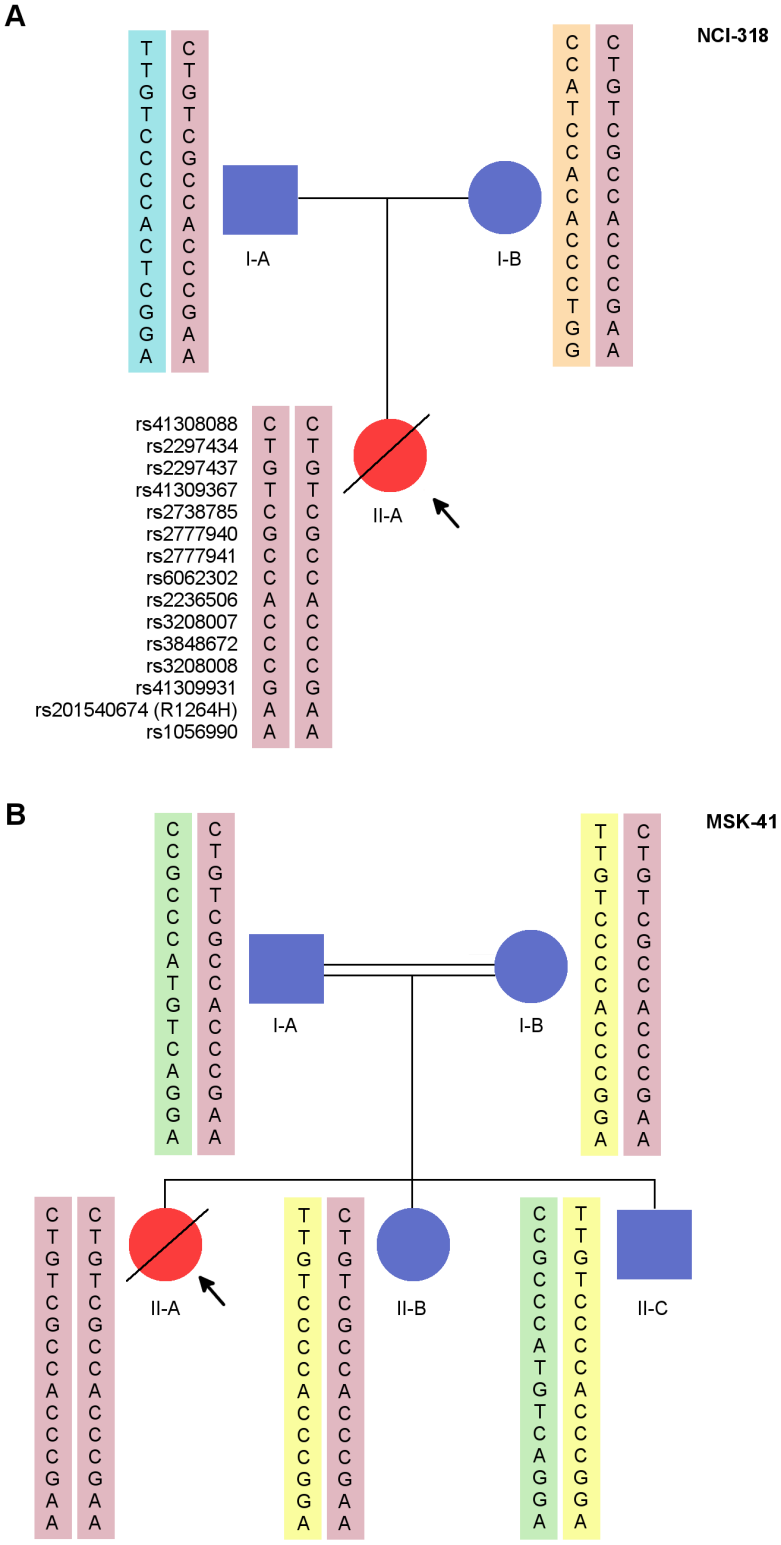
NCI-318 and MSK-41 pedigrees with *RTEL1* mutation and shared risk haplotype. NCI-318 (A) and MSK-41 (B) pedigrees are shown. Red symbols indicate affected individuals. The pink rectangles indicate the shared haplotype between the pedigrees. Each other colored rectangle indicates a unique haplotype.

**Figure 2:**
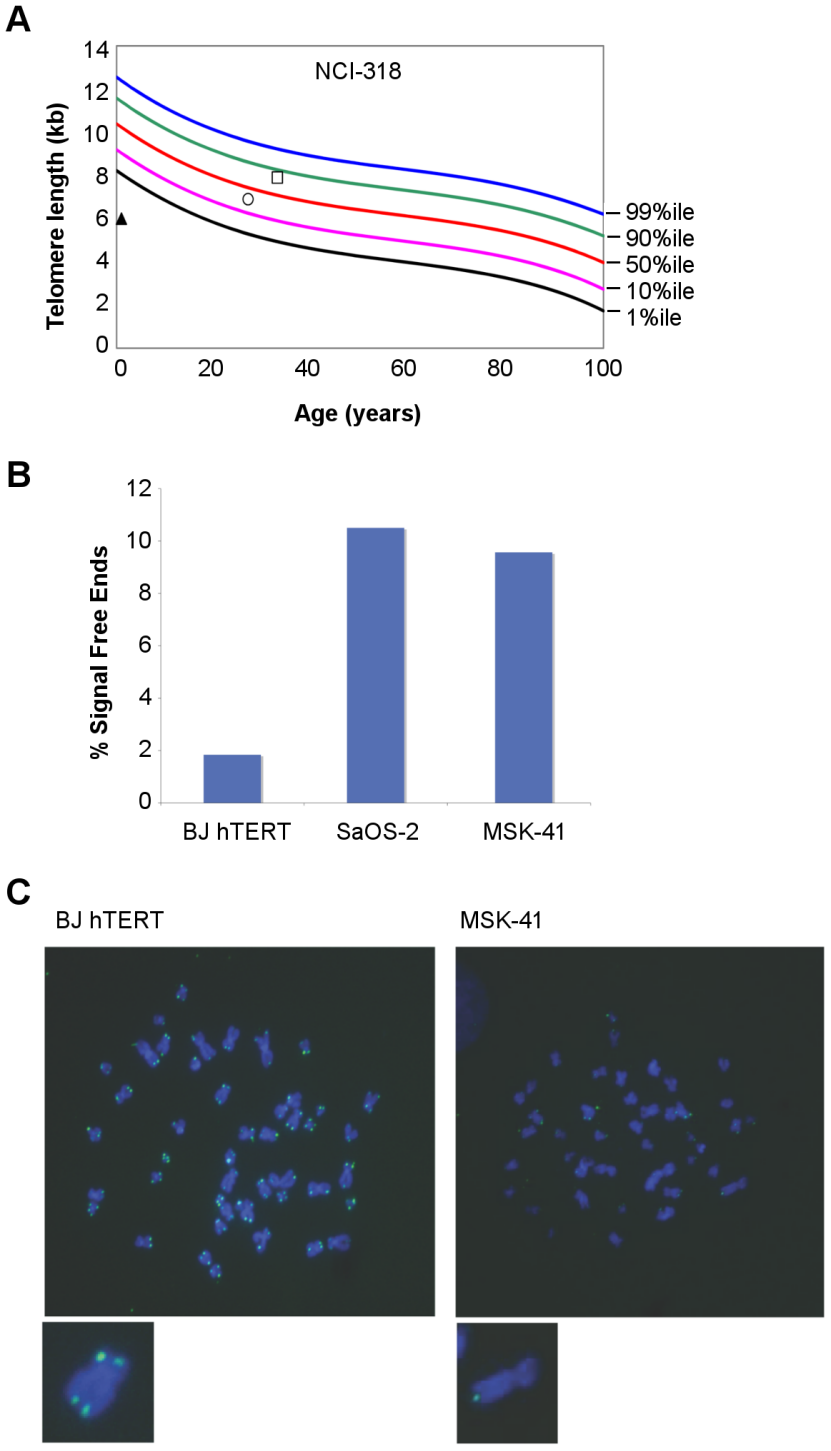
Telomere length is altered in individuals with *RTEL1^R1264H^*. (A) Primary lymphocyte telomeres in family NCI-318 were measured by flow cytometry with fluorescent *in situ* hybridization (FISH) [3]. The proband is indicated by a triangle, the mother by a circle, and the father by a square. (B) Telomere FISH analysis of MSK-41 hTERT-immortalized fibroblasts revealed extreme telomere length heterogeneity. Quantitation of chromatids lacking detectable telomeric signal is shown. BJ hTERT, a normal hTERT-immortalized fibroblast line, and SaOS-2, an osteosarcoma cell line that relies on recombination-based telomere maintenance (ALT), are presented for comparison. (C) Representative metaphase spreads of MSK-41 and BJ hTERT are shown.

The female proband, NCI-318-1 (family NCI-318) was born prematurely at 32 weeks gestation due to placental clots (Table 1, Figure 1A). Her parents were unrelated and of AJ ancestry. She was small for age and had poor postnatal growth. At 6 months of age she developed recurrent, chronic diarrhea and rectal prolapse. An extensive evaluation for allergic and infectious etiologies was negative. At 11 months of age, a colonoscopy showed severe colitis with evidence of apoptosis in the colonic epithelium. A concurrent immunologic evaluation showed low total B cells (CD 20+) at 14 cells/mm^3^, NK cells at 65 cells/mm^3^, and CD8+ T cells were 487 cells/mm^3^ (normal tenth percentiles are 1,310 cells/mm^3^, 360 cells/mm^3^, and 2,100 cells/mm^3^, respectively [10]), and her mitogen studies were abnormal. Her IgG was low at 26 mg/dL, IgA<5 mg/dL, IgM 29 mg/dL (lower limits of normal for age are 453 mg/dL, 20 mg/dL, and 19 mg/dL, respectively). Chromosome breakage studies were not consistent with Fanconi anemia. Subsequent testing identified peripheral blood telomere length as very short for her age (Figure 2A). An MRI of her brain showed cerebellar hypoplasia. Based on her clinical history and very short telomeres, she was diagnosed with the HH variant of DC. Genetic testing for *TERT, TERC, TINF2, NOP10, NHP2*, and *WRAP53* was negative. She died due to complications following bone marrow transplant at two years of age. The mother and father are both clinically healthy, and their telomeres are normal (30 percentile and 70 percentile for age, respectively) (Figure 2A).

**Table 1:** Clinical characteristics of families with *RTEL1* mutations.

Family	Participant	Age at Study Entry (years)	Clinical Features
NCI-318	Female Proband, NCI-318-1	1.4	Findings consistent with HH including, prematurity, IUGR, microcephaly, cerebellar hypoplasia, developmental delay, marked short stature, failure to thrive, severe enteropathy, severe B and NK cell immunodeficiency, low IgG, thrombocytopenia, very short telomeres for age, died due to MUD HSCT-related complications
NCI-318	Mother, NCI-318-2	27	Healthy
NCI-318	Father, NCI-318-3	33	Healthy
MSK-41	Female Proband	0.7	Features consistent with HH including, IUGR, microcephaly, developmental delay, marked short stature, failure to thrive, severe enteropathy, severe B and NK cell immunodeficiency, hypogammaglobulinemia, died before engrafting post mis-matched related HSCT
MSK-41	Sister	N/A	Preterm, IUGR, microcephaly, developmental delay, marked short stature, failure to thrive, severe B and NK cell immunodeficiency, hypogammaglobulinemia, died due to infection
MSK-41	Brother	16	Healthy
MSK-41	Sister	12	Healthy
MSK-41	Brother	10	Healthy
MSK-41	Brother	9	Healthy
MSK-41	Mother	37	Healthy
MSK-41	Father	38	Healthy

Abbreviations: DC, dyskeratosis congenita; HH, Hoyeraal Hreidarsson syndrome; BMF, bone marrow failure; IUGR, intra-uterine growth retardation; MUD HSCT, matched-unrelated donor hematopoietic stem cell transplantation; N/A, not applicable.

#### MSK-41 Patient

The female proband, MSK-41, was born prematurely at 29 weeks gestation with IUGR, weight 615 grams (Table 1). Her parents, both of whom are healthy, are consanguineous and of AJ ancestry (Figure 1B). She had poor postnatal growth, gastroesophageal reflux, and vesicouretal reflux. She was evaluated for a potential immunodeficiency at the referring institution, as an older sister also born prematurely with IUGR had died at 15 months of age of systemic adenovirus prior to the family's enrollment in the study. The sister had microcephaly, developmental delay, failure to thrive, severe B and NK cell immunodeficiency, and hypogammaglobulinemia. At 6 months of age, MSK-41 developed an upper respiratory tract infection due to influenza and at 7.1 months of age, she was hospitalized for fever, but had negative cultures. At 7.2 months of age, she was readmitted for fever and diarrhea, and was found to have high-grade cytomegalovirus (CMV) viremia. She was placed on anti-viral therapy and referred to Memorial Sloan-Kettering Cancer Center for evaluation for transplant. Although her total white blood cell (WBC), hemoglobin, and platelet counts were normal prior to the development of CMV viremia, she developed count suppression secondary to the virus and anti-viral therapy. Her initial immunologic evaluation showed mildly decreased numbers of circulating CD4+ and CD8+ T-cells, low NK-cell numbers, and low B-cell numbers for age. She subsequently developed progressive T-, B-, and NK-cell lymphopenia and hypogammaglobulinemia, and she lacked specific B-cell responses to vaccines administered at 2 and 4 months of age. Her T-cell function waxed and waned but at 8.5 months of age, she had a normal T-cell response to phytohemagglutinin and allogeneic cells, but lacked response to Candida or CMV. CT scan and subsequent MRI of the head showed normal sized ventricles and sulci, and the gray-white matter differentiation was considered normal for her gestational age. She was neurologically normal for her gestational age until she developed the CMV infection. Laboratory work-up revealed normal levels of adenosine deaminase (ADA) and purine nucleoside phosphorylase (PNP), and absence of mutations in genes associated with immunodeficiency including *RAG1*, *RAG2*, *CD3D*, *CD3E*, and *DCLRE1C*. Although lymphocyte defects and impaired growth can be caused by inherited defects in DNA repair genes, DNA sequencing did not reveal evidence of DNA ligase IV deficiency, Cernunnos defects, ataxia telangiectasia, Nijmegen breakage syndrome, Bloom syndrome, or Fanconi anemia. She died 41 days following a T-cell depleted HLA-mis-matched related stem cell transplant without evidence of engraftment. There are four additional healthy siblings in the family: three brothers and one sister.

#### Sequence Analyses

DNA from the family trio NCI-318 was analyzed by whole exome sequencing (WES). Variants identified by WES were evaluated in AD, AR, and XLR inheritance models (Tables S1 and S2). We also ensured that there was adequate coverage of known DC genes, including the recently-discovered DC-associated gene *CTC1* [11] and the non-protein-coding *TERC* locus. After filtering out common variants (Table S1), the top candidate variants that fit the most likely inheritance model were validated by an orthogonal sequencing technology (Materials and Methods). While we found variants in several telomere maintenance and DNA damage repair genes (Table S3), most were heterozygous in the proband and her father. Given that the father had longer-than-average telomeres for his age and was clinically healthy, we proposed that an autosomal recessive model was more likely than a paternal autosomal dominant one. An analysis of rare AR variants revealed three candidate single nucleotide variants (SNVs) (Table S2), of which *RTEL1*, an evolutionarily conserved helicase involved in telomere replication and stability, was the most biologically plausible. The proband was homozygous for a mutation (g.20:62326972G>A (hg19), hereafter referred to as *RTEL1^R1264H^*), and each parent was a heterozygous carrier of this mutation (Figure 1A). We did not observe any compound heterozygous variants in this family that met our filtering criteria.

Fibroblast DNA from MSK-41 underwent targeted sequencing of approximately 300 genes involved in the DNA damage response or implicated in maintaining genome stability. Amongst those candidate genes, the only variant found was a homozygous *RTEL1^R1264H^* mutation (Figure 1B). Importantly, except for *RTEL1*, most other candidate variants found in NCI-318 by exome sequencing were not recapitulated in MSK-41 (Table S2). Follow-up sequencing indicated that both the mother and father of MSK-41 were heterozygous carriers of *RTEL1^R1264H^*.

**Figure 3:**
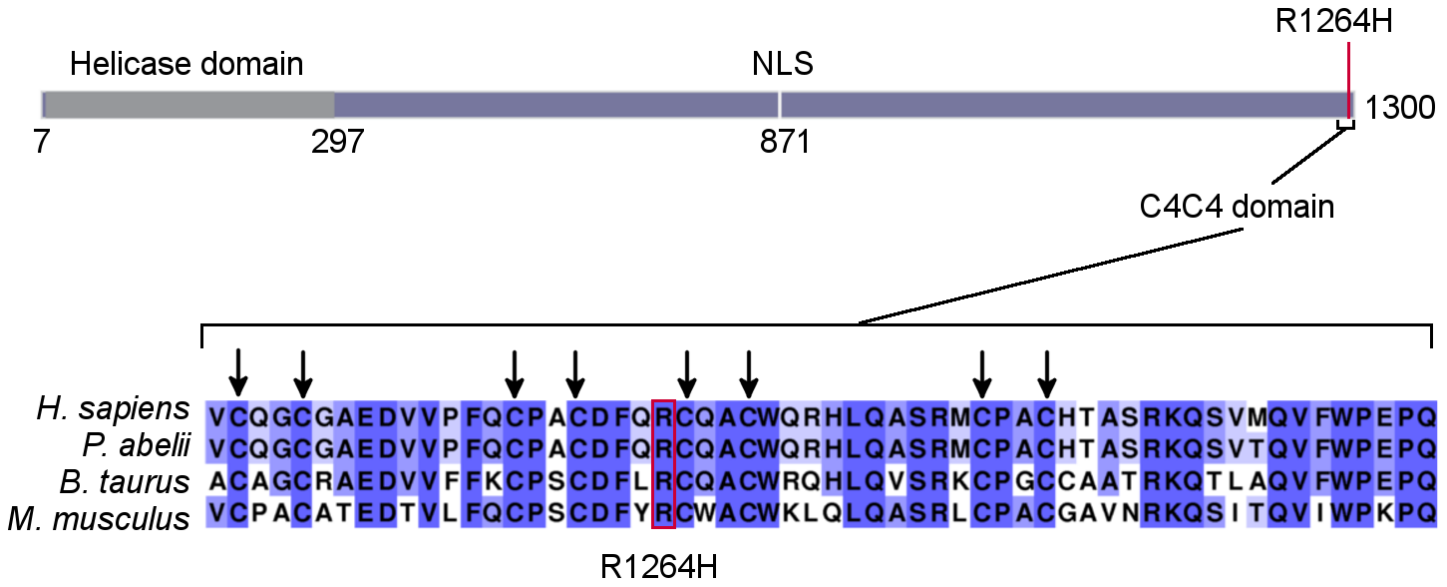
*RTEL1^R1264H^* affects a putative conserved C4C4 domain. As displayed on the schematic (representing ENSP00000353332), the RTEL1 mutation is at the C-terminus of the protein, distal to the helicase domain. The affected amino acid is in a putative C4C4 domain. All eight key cysteines and R1264 are conserved in human, orangutan, cattle, and mouse sequences. Higher percent identity at a given amino acid position is indicated by a deeper purple color.

The *RTEL1^R1264H^* mutation affects three *RTEL1* protein-coding isoforms (UniProt identifiers Q9NZ71-6, Q9NZ71-2 and Q9NZ71-5, in which the affected amino acid is R509; Ensembl IDs ENST00000360203462/ENSP00000353332, ENST00000318100/ENSP00000322287, and ENST00000370003/ENSP00000359020) and encodes a previously undefined C4C4 RING finger domain (Figure 3). This domain is characterized by a specific pattern of cysteine residues conforming to the consensus sequence Cx_2_C x_9_ Cx_2_C x_4_ Cx_2_C x_10_ Cx_2_C. Despite the somewhat conservative amino acid change, R1264 is highly conserved (Figure 3), and is centrally located within the putative C4C4 Zn2+ coordination domain; therefore, the R1264H change is likely to exert a substantial impact on RTEL1 function. *In silico* prediction algorithms (SIFT, PolyPhen-2, and Condel) indicate that this amino acid substitution is likely to be damaging to the protein. The *TNFRSF6B* gene is adjacent to the *RTEL1* locus, and *RTEL1* exon 34 sequences are present in non-coding exons of the *TNFRSF6B* transcript as well as in a non-coding *RTEL1*-*TNFRSF6B* read-through transcript, raising the possibility that the mutation may also affect *TNFRSF6B* expression. However, western blotting of MSK-41 whole cell extracts indicated no change in the TNFRSF6B levels (Figure S1), arguing that the effects of the mutation are confined to *RTEL1*.

#### Haplotype Analysis

An analysis of 15 common SNPs in the 1000 Genomes European populations distributed over the *RTEL1* locus indicated low linkage disequilibrium in the ∼34,000 bases surrounding the g.20:62326972G>A mutation that encodes *RTEL1^R1264H^*. This results in several haplotypes in healthy populations within the 1000 Genomes Project [12]. The carrier parents and affected individuals in our families were the only individuals we found to have haplotypes containing the G>A mutation (compared with 378 of 1000 Genomes samples of European ancestry). Sanger sequencing was performed to determine the genotypes of 12 common single nucleotide polymorphisms in all the available family members of both families. These included the trio from NCI-318 and five individuals from MSK-41 (see pedigree, Figure 1A and 1B). Three SNPs that were in strong linkage disequilibrium (r^2^ = 1) with the genotyped SNPs were also included in the analysis. These polymorphisms were chosen to be within the region chr20:62,292,868–62,327,449 (hg19) that encompasses *RTEL1* exons 4 through 35, a region that also includes the *RTEL1^R1264H^* mutation. The probands in both families were homozygous for the mutation and all genotyped SNPs (Figure 1A and 1B). Haplotypes were reconstructed based on allele sharing in the unaffected siblings and parents. No recombinants were seen in either family and the segregating risk haplotype was identical in NCI-318 and MSK-41. In MSK-41, the unaffected individuals II-B and II-C inherited one copy and no copies of the risk haplotype containing the mutant allele, respectively. Thus we show that the R1264H variant is carried on a common haplotype, likely from a common AJ founder. Notably, the variant is not seen in the publically available data on approximately 9,000 individuals (ESP 6500 or the 1000 Genomes); however, dbSNP 137 shows the entry rs201540674 with a minor allele frequency (MAF) of 0.002 in a population of approximately 600 individuals of European descent. The combined data from these three sampled populations suggests a very low carrier frequency of approximately 1 in 9,600 individuals (MAF ∼0.0001). Because this is a recessive allele, the disease-associated genotype frequency would then be approximately 1 in 100 million in the general population, which is consistent with the low prevalence of this disorder.

#### Cellular Phenotype

As expected for DC patients, primary lymphocytes from the NCI-318 proband (Figure 2A) and the MSK-41 hTERT-immortalized fibroblast line exhibited clear indications of defects in telomere maintenance (Figure 2B and 2C). Notably, extreme heterogeneity in telomere length was evident in MSK-41 cells despite immortalization with hTERT. The frequency of chromatid ends lacking telomeric FISH signal in MSK-41 cells was approximately 10%, approaching that seen in SaOS2, a cell line with the alternative lengthening of telomeres (ALT) phenotype [13]. A similar outcome was observed upon inactivation of the *RTEL1* gene in murine embryonic fibroblasts (MEFs) [14], indicating that the telomere defects observed are likely attributable to a decrement in RTEL1 function due to the *RTEL1^R1264H^* mutation.

**Figure 4:**
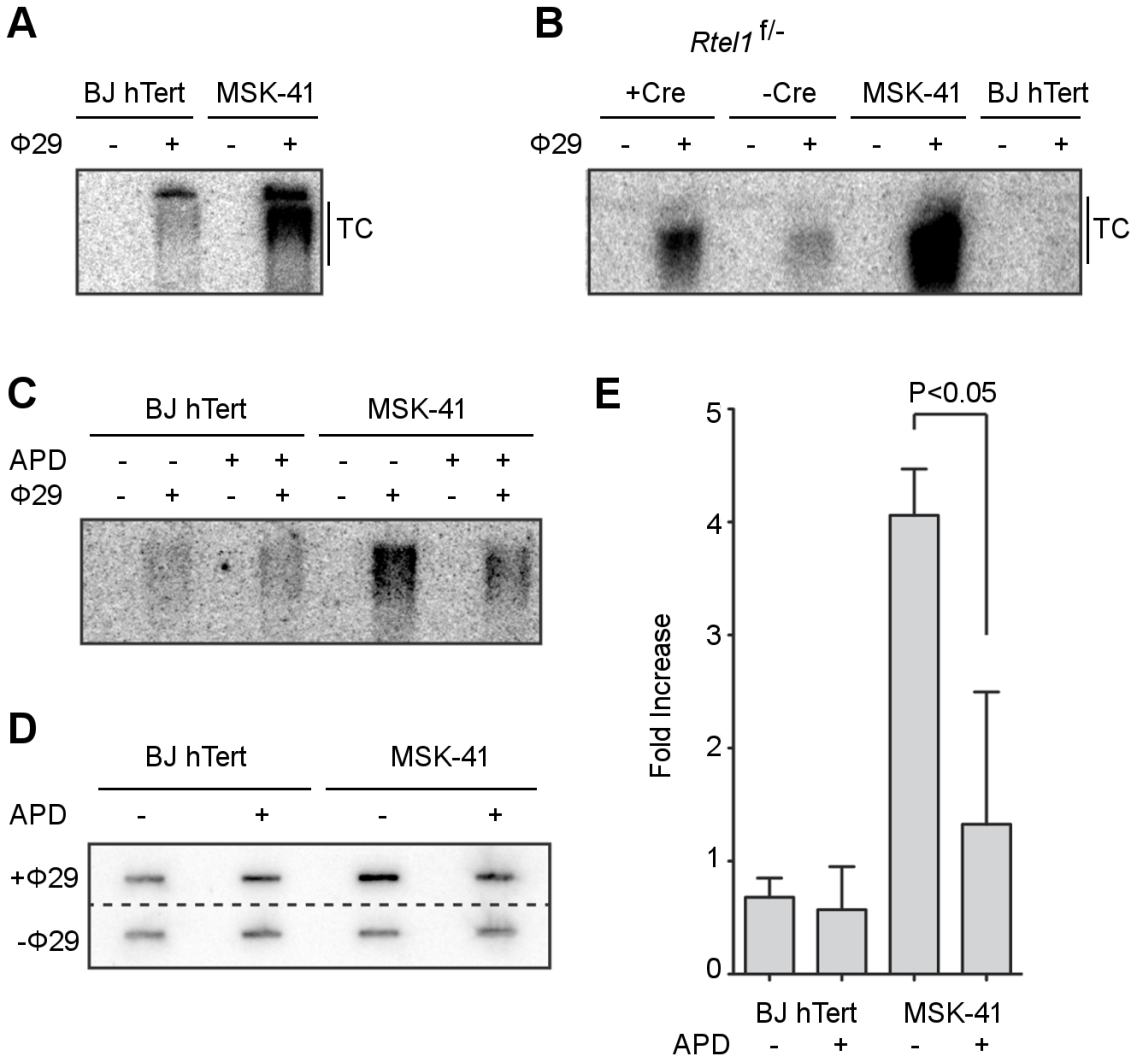
Inhibiting DNA replication blocks T-circle formation in MSK-41 *RTEL1^R1264H^* cells. (A) Phi29-dependent T-circles in BJ hTERT and MSK-41. (B) Phi29-dependent T-circles in RTEL1 floxed/- MEFs ± Cre, BJ hTERT and MSK-41. (C) Phi29-dependent T-circles in BJ hTERT and MSK-41 ± aphidicolin (APD; 5 µM). (D) Dot blot of the Phi29-dependent T-circles in BJ hTERT and MSK-41 ± aphidicolin (APD; 5 µM). (E) Quantification of the fold increase in intensity of Phi29-dependent T-circles in the different cell lines subjected to the indicated treatments. Intensity mean and standard deviation were calculated over two independent experiments; statistical analysis (one-way ANOVA) was calculated with Prism (GraphPad).

Loss of telomeric sequence upon conditional deletion of *RTEL1* in MEFs is accompanied by the formation of extrachromosomal T-circles [14]. T-circles are proposed to arise in RTEL1-deficient cells when the DNA replication machinery collides with the T-loop structure that would otherwise be dismantled by RTEL1 to permit replication of the chromosome end. Therefore, we examined the MSK-41 hTERT-immortalized cell line for the presence of T-circles to determine whether the *RTEL1^R1264H^* mutant phenocopied RTEL1 deficiency in this regard. T-circles are detected by annealing a telomere-specific primer to denatured genomic DNA, followed by treatment with Phi29 DNA polymerase. In this setting, circular DNA is amplified by a rolling circle mechanism, whereas linear telomeric DNA is not [14], [15]. When subjected to the amplification assay, genomic DNA from MSK-41 cells gave rise to levels of T-circles approximating those seen upon conditional activation of *RTEL1* in mouse embryonic fibroblasts (Figure 4A and 4B). This suggests that in cells bearing the *RTEL1^R1264H^* mutation, telomeres are compromised due to an inability to appropriately resolve the T-loop structure.

In further support of this model, the formation of T-circles depends on an intact DNA replication process. MSK-41 hTERT cells exhibited four-fold higher levels of T-circles compared with BJ hTERT control cells (Figure 4C, 4D, 4E); however, when DNA replication was inhibited by the addition of 5 µM aphidicolin, the T-circle-derived signal in MSK-41 cells was significantly reduced, as inferred from electrophoretic analysis and slot blotting of Phi29-treated genomic DNA. Collectively, these data strongly support the interpretation that the *RTEL1^R1264H^* mutation impairs the functions of RTEL1 at the telomere.

**Figure 5:**
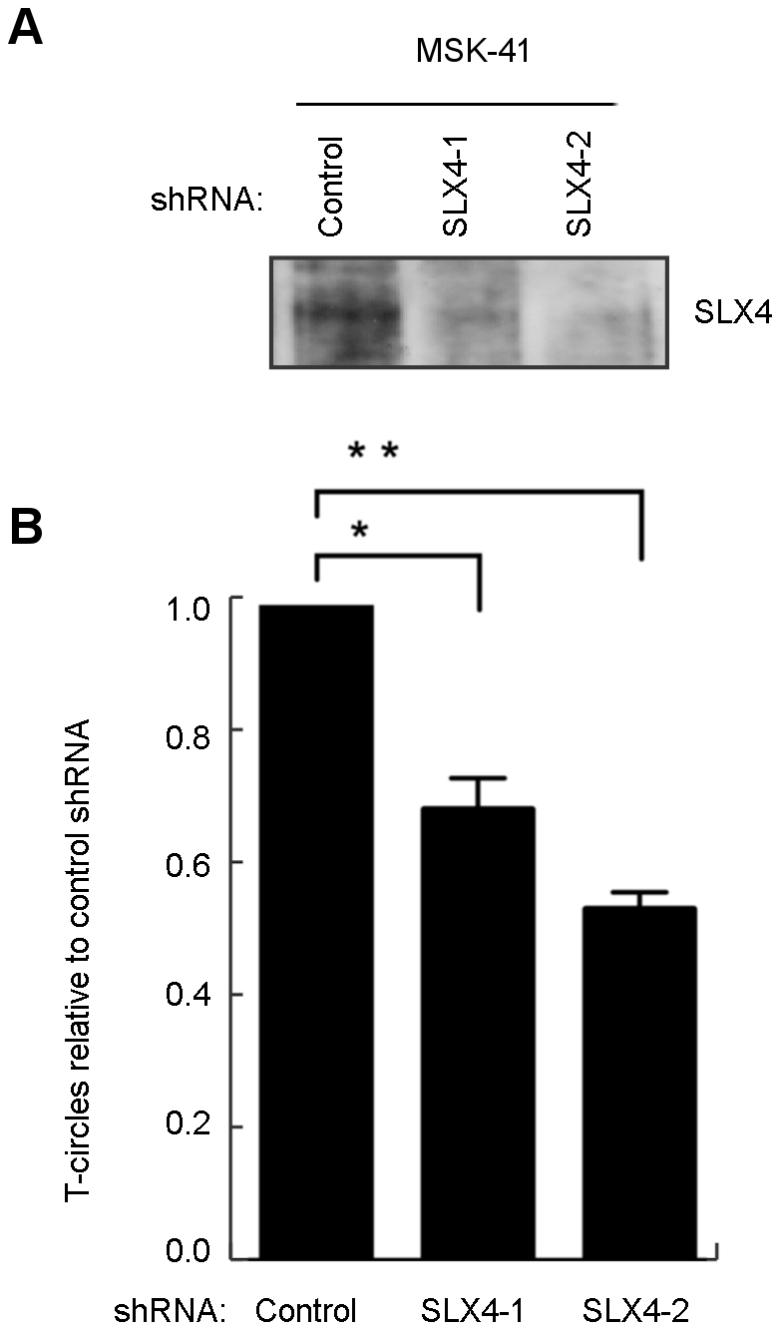
T-circle formation in MSK-41 cells is dependent on SLX4. (A) Two shRNAs (SLX4-1 and SLX4-2) were used to knockdown SLX4 expression. (B) T-circle formation was measured in the MSK-41 SLX4 knockdown strains relative to MSK-41 with a control shRNA. Mean and standard deviation were calculated from two independent experiments. *P<0.05, **P<0.01 by unpaired two-tailed t-test.

As reported previously, T-circle formation in RTEL1-deficient cells is dependent on the nuclease SLX4, and knockdown of SLX4 in an RTEL1-deficient background results in a rescue of the telomere loss phenotype [14]. To determine whether the *RTEL1^R1264H^* mutation impeded appropriate resolution of T-loops, we reduced the expression of SLX4 in MSK-41 cells. We performed transient knockdown experiments using two different short hairpin RNAs (shRNAs) targeting SLX4 in the MSK-41 hTERT cell line (Figure 5A). Both shRNAs result in efficient knockdown of SLX4 (Figure 5A) and suppression of T-circle formation (Figure 5B); the extent of suppression correlates with the degree of knockdown of SLX4. This confirms that the *RTEL1^R1264H^* mutation has a deleterious effect on RTEL1 function. Stable expression of the SLX4 shRNAs in MSK-41 cells did not achieve sufficient knockdown of SLX4 (data not shown), and therefore we were unable to assess the effect on telomere loss in this cell line.

**Figure 6:**
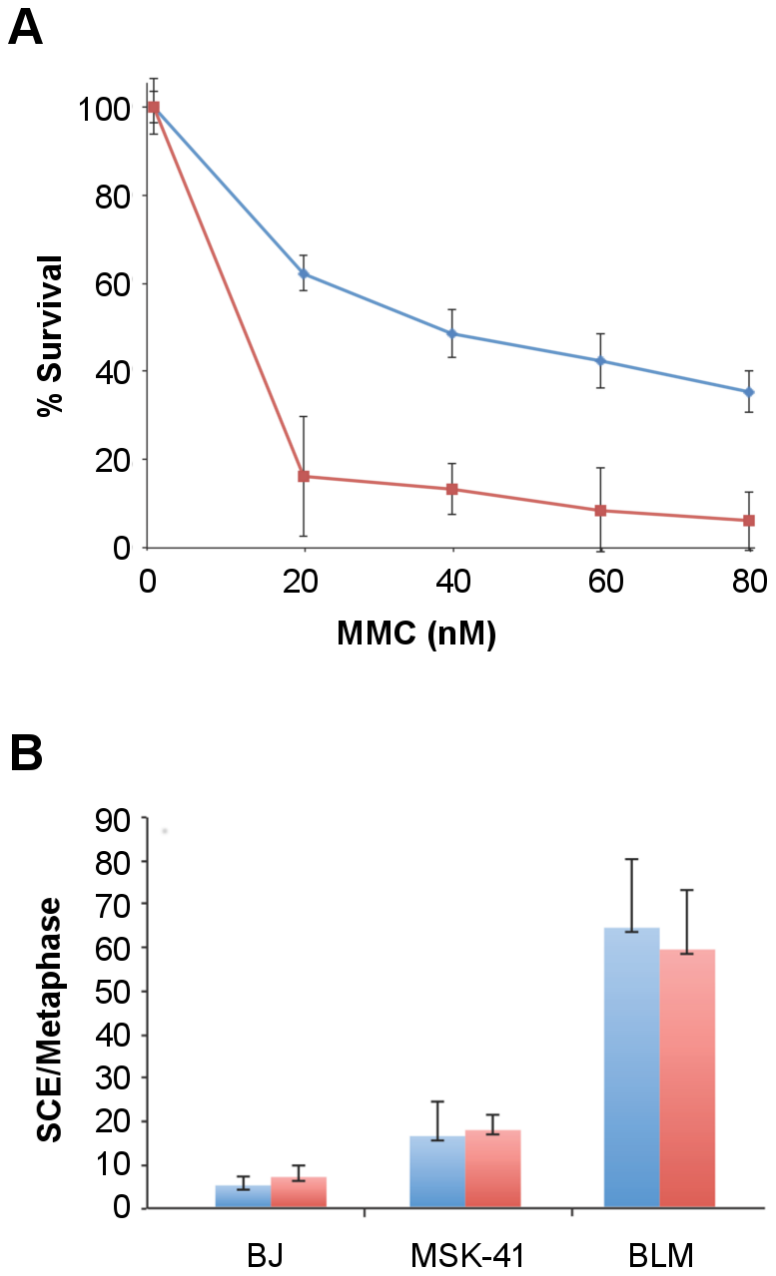
MSK-41 cells are hypersensitive to DNA damage and experience elevated levels of sister chromatid exchange. (A) BJ hTERT (blue line) and MSK-41 cells (red line) were treated at the indicated doses of mitomycin C (MMC) for 24 hours, and colony formation was scored 14 days post-treatment. Formation of at least 50 colonies was required at each dose for the experiment to be considered valid. (B) Spontaneous (blue) and MMC-induced (red) sister chromatid exchanges were visualized by Giemsa staining; the number of exchanges per metaphase is shown. Cells were cultured in 20 µM BrdU for 40 hours, with treatment with 25 ng/mL MMC for the final 24 hours.

Similar to its proposed role at T-loops, RTEL1 mediates dismantling of displacement loops, or D-loops, which are formed as intermediates in homology-directed DNA double strand break (DSB) repair at telomeres and throughout the genome [16]. This function prevents the execution of inappropriate recombination events, and is proposed to thereby suppress deleterious genome rearrangements and enforce the orderly repair of DSBs [17]. To determine whether non-telomeric functions of RTEL1 were affected by the *RTEL1^R1264H^* mutation, we assessed the sensitivity of MSK-41 hTERT cells to the DNA crosslinking agent mitomycin C (MMC). Cells were subjected to MMC for 24 hours (20–80 nM), and plated for colony formation, with BJ hTERT serving as the wild-type control. We observed a modest (8–10 fold) increase in sensitivity to MMC at all doses, indicating that the repair of DNA crosslinks was impaired in the *RTEL1^R1264H^* mutant (Figure 6A).

In addition to MMC sensitivity, we observed an increase in the spontaneous levels of sister chromatid exchanges (SCE) in MSK-41 hTERT cells, indicating an increase in genomic instability in the presence of the *RTEL1^R1264H^* mutation. SCEs were observed in 18% of MSK-41 metaphase spreads, approximately a two-fold increase over the levels seen in BJ hTERT control cells, but 3-fold less frequently than observed in a Bloom Syndrome fibroblast line (Figure 6B). MMC treatment had no effect on SCE levels in any of the genotypes observed. Although the SCE phenotype in MSK-41 cells is less severe than observed in Bloom Syndrome cells, the increased levels are likely to reflect a reduction in the anti-recombination functions of the *RTEL1^R1264H^* gene product. Hence, both the telomeric and non-telomeric functions of RTEL1 are affected by the *RTEL1^R1264H^* mutation. However, the general DNA damage repair phenotype in MSK-41 cells is not as severe as that of cells derived from a patient with Bloom Syndrome, a disorder marked by primary dysfunction in the DNA damage repair machinery.

## Discussion

This study demonstrates the clinical and molecular consequences of homozygous autosomal recessive mutations in *RTEL1*. We identified two families with children who had HH, were of AJ ancestry, and had the same homozygous *RTEL1^R1264H^* mutations. These data provide further evidence that defects in RTEL1 function can lead to clinical phenotypes consistent with the HH variant of DC [6]. Our molecular analyses indicate that the homozygous *RTEL1^R1264H^* mutation results in short, heterogeneous telomeres. Additionally, cell lines bearing this mutation produce excess extrachromosomal T-circles, but only in the presence of functioning DNA replication machinery. RTEL1 is proposed to resolve T-circles to enable proper telomeric replication; in the absence of this activity, T-loops are inappropriately resolved as a circle when encountered by the replication machinery, resulting in a shortened telomere [18]. T-circle formation in the presence of *RTEL1^R1264H^* is SLX4-dependent, similar to T-circle formation in RTEL1-deficient cells [14].

RTEL1 also aids in suppressing inappropriate recombination throughout the genome. We have shown that the *RTEL1^R1264H^* mutation results in a modest enhancement in sensitivity to DNA damage, as well as an increase in SCE, indicating that the *RTEL1^R1264H^* mutation impairs both telomeric and non-telomeric aspects of RTEL1 function.

The fact that both the probands were homozygous for the identical risk haplotype suggests that there is an ancestral haplotype that is shared by parents in both families (Figure 1A and 1B). We were able to reconstruct the haplotype based on the genotypes obtained using Sanger sequencing. This haplotype was also seen without the mutation in 14/378 (TSI/GBR/FIN) samples of EUR ethnicity in the 1000 Genomes data. Together with the occurrence of the risk haplotype in the two families with AJ ethnicity, the evidence supports the interpretation that this mutation is confined to EUR populations and is most likely an AJ founder mutation. We have not extended the 34 kb haplotype further since the number of individuals with this rare recessive disorder in our study is too small to investigate the age of the mutation based on haplotypes and population history.

We and others recently reported that AD nonsense *RTEL1* mutations are present in HH and that an additional missense mutation in the helicase domain further exacerbates the clinical and telomere length phenotype, while the presence of only a single missense mutation in the helicase domain resulted in a less clinically severe phenotype [6], [7].[8] The current study provides important insight into the function of the C-terminal end of the human RTEL1 protein. *RTEL1* deficiency confers embryonic lethality in mice [19], suggesting that the R1264H allele is hypomorphic. As is the case for the two families described here, hypomorphs are usually recessive; for example, AR partial loss-of-function mutations in *FANCD2* cause Fanconi anemia and AR *LIG4* mutations result in Ligase IV syndrome [20], [21]. Furthermore, this mutation is distal to the RTEL1 helicase domain, and is thus unlikely to directly affect enzymatic activity. Nevertheless, the phenotypic impact of *RTEL1^R1264H^* at the cellular level was pronounced.

The *RTEL1^R1264H^* mutation falls within exon 34, which encodes a predicted C4C4 RING domain of RTEL1, lying downstream of a putative PIP box. Many RING domain-containing proteins are E3 ubiquitin ligases that interact with E2 ubiquitin-conjugating enzymes through their RING domains. BRCA1, MDM2, and Parkin are all examples of RING domain-containing proteins that are involved in human disease [22]. The putative RTEL1 RING domain is distant from the helicase domain, suggesting that the *RTEL1^R1264H^* mutation may affect the RING domain while leaving the helicase activity intact. Given the severity of the clinical and cellular phenotypes of this mutation, the data suggest that this domain exerts a significant influence on the biological function of RTEL1. Further analysis of this domain to define the mechanism(s) of its influence is ongoing.

These findings, together with the recent report that non-coding SNPs in *RTEL1* have been found to be associated with susceptibility to high-grade glioma [23]–[25], broadly implicate the *RTEL1* locus in human cancer susceptibility. Given the cellular phenotypes of DC/HH and those reported here, the clinical features of DC are likely sequelae of defects in maintenance and functions of the telomere.

We have demonstrated that the *RTEL1^R1264H^* mutation affects both the telomeric and non-telomeric functions of RTEL1. Individually, proteins involved in either telomere maintenance or DNA repair can result in immunodeficiency when perturbed: DC is an example of the former, and Bloom syndrome of the latter. The patients described here exhibit severe immunodeficiency, which may be the result of a mutation affecting both of these pathways. However, future studies are required to better understand this observation.

## Materials and Methods

### Ethics Statement

This research was approved by the Institutional Review Boards (IRB) of the National Cancer Institute and Memorial Sloan Kettering Cancer Center. All participants or their parents signed IRB-approved informed consent forms.

### Patients

Patient NCI-318 and her family were participants in an IRB-approved longitudinal cohort study at the National Cancer Institute (NCI) entitled “Etiologic Investigation of Cancer Susceptibility in Inherited Bone Marrow Failure Syndromes” (NCI 02-C-0052, ClinicalTrials.gov Identifier: NCT00027274). In this study, patients and their family members complete questionnaires and undergo thorough clinical evaluations at the NIH Clinical Center [2]. Telomere length was measured by flow cytometry with fluorescent *in situ* hybridization (flow FISH) in leukocytes [26].

THE MSKCC proband was ascertained on IRB-approved protocol 95-091 entitled “Collection of Hematopoietic Progenitor Cell and/or Blood Samples From Patients For Research Studies.” Other family members consented to germline testing in the Clinical genetics Service, as well as MSKCC 93-102 “Ascertainment of Peripheral Blood or Saliva Samples for Genetic Epidemiology Studies of Familial Cancers,” as well as a specific consent for the novel homologous recombination gene described in this report.

### Exome Sequencing, Analysis, and Variant Prioritization

Whole exome sequencing for family NCI-318 was performed at the NCI's Cancer Genomics Research Laboratory as previously described [6].

Reads were aligned to the hg19 reference genome using Novoalign software version 2.07.14 (http://www.novocraft.com), Picard software version 1.67 (http://picard.sourceforge.net/) and the Genome Analysis Toolkit (GATK, http://www.broadinstitute.org/gatk/) [27]. Variant discovery, genotype calling, and annotation were performed as described [6] using data from the UCSC GoldenPath database (http://hgdownload.cse.ucsc.edu/goldenPath/hg19/database/), the ESP6500 dataset from the Exome Variant Server, NHLBI Exome Sequencing Project (ESP), Seattle, WA (http://evs.gs.washington.edu/EVS/) (accessed August 2012), the Institute of Systems Biology KAVIAR (Known VARiants) database (http://db.systemsbiology.net/kaviar/) [28], the National Center for Biotechnology Information dbSNP database (http://www.ncbi.nlm.nih.gov/projects/SNP/) [29] build 137, and the 1000 Genomes (http://www.1000genomes.org/) [12]. Variants were also annotated for their presence in an in-house database consisting of over 700 whole exomes that were sequenced in parallel with our DC families. Variants within each family were filtered and categorized as indicated in Table S1.

### *RTEL1* Targeted Sequencing

Validation of exome sequencing findings in the NCI-318 trio was performed by sequencing coding exons of *RTEL1*. Primer sequences are shown in Table S4. All samples were amplified using KAPA2 RobustHotstart Readymix (2×) (Kapa Biosystems, Johannesburg, South Africa) and the following cycling conditions: 3 min at 95°, followed by 30 cycles of 15 sec at 95°, 15 sec at 60°, 15 sec at 72°, followed by 10 min at 72°. Amplicons were purified using Agencourt's Ampure XP beads, then libraries were constructed and barcoded using the Ion Xpress Plus Fragment Library Kit (Life Technologies, Carlsbad, CA, USA). DNA tagged beads were generated for sequencing using Life Technologies' OneTouch and run on an Ion 316 chip on the Ion PGM Sequencer (Life Technologies). The default TMAP aligner and variant caller was used to generate a variant list per sample.

### MSK-41 Sequencing

Targeted resequencing of DNA damage response genes was instrumental in the discovery of the RTEL1 mutation at MSKCC. Genomic enrichment via microfluidic PCR was conducted using the primer pool from Raindance Technologies [30]. Resulting libraries were prepared for sequencing using the SOLiD 4 sequencer (Life Technologies, Carlsbad). Read alignment and base-calling was done using the ABI Bioscope software with parameters optimal for targeted resequencing. Reads were filtered for mapping quality. *RTEL1* contained the most biologically relevant non-synonymous exonic variant. MSK-41 was included in a panel of 24 cell lines in which targeted DNA sequencing of approximately 300 DNA damage response genes (including *RTEL1*) was carried out (see methods [13]).

### *In silico* Analysis

PolyPhen-2 [31] (http://genetics.bwh.harvard.edu/pph2), SIFT [32] (http://sift.jcvi.org), and Condel [33] (http://bg.upf.edu/condel/home) were used to predict the severity of RTEL1 amino acid substitutions. Multiple sequence alignments were generated for homologous RTEL1 protein sequences using T-Coffee [34] (www.tcoffee.org) to evaluate conservation. Alignments were generated with NCBI Reference Sequence, GenBank or Ensembl proteins ENSP00000353332 (*Homo sapiens*), NP_001124929.1 (*Pongo abelii*), NP_001091044.1 (*Bos taurus*), and EDL07405.1 (*Mus musculus*).

### Telomere FISH Analysis

Telomere FISH was performed as described [35]. Images were captured at 100× magnification, with precisely the same exposure time for each genotype (MSK-41 hTERT and BJ hTERT). Sensitivity (gain) is increased to saturation, and chromosome ends for which there still appears no signal are scored as signal-free ends. The heterogeneity observed in Figure 2C was reproducible over several experiments, and with different probes (data not shown).

### Genomic DNA Extraction and T-Circle Amplification

Cells were collected from 2 to 3 10 cm plates at 70% confluence for each condition. Genomic DNA extraction was performed as described [36]. DNA was double digested by AluI/HinfI restriction enzymes overnight before starting TCA assay and then Southern Blot as described [37] with minor modifications to Phi29 DNA polymerization (MBI Fermentas) with a mammalian telomeric primer and a mammalian telomeric probe for hybridization. Blot images were captured and quantified with Storm 840 scanner and ImageQuant TL software (Amersham Biosciences).

Sister chromatid exchange analysis and telomere FISH were carried out as described previously [35]. Mitomycin C sensitivity assays were as described [38].

### SLX4 Knockdown

To detect SLX4 levels in the various knockdown conditions, we immunoprecipitated SLX4 (1.5 mg protein lysate, 10 µg of antibody) with rabbit polyclonal antibody (A302-269A) followed by western blotting with polyclonal rabbit antibody A302-270A. Both antibodies were from Bethyl. T-circles were detected and quantified as previously described [14].

### Cell Culture

Immortalized conditional RTEL1F/- MEFs were as previously described [14] and were cultured in DMEM containing 10% fetal bovine serum. Cre recombination was carried out with Ad5-CMV-Cre adenovirus (Vector Biolabs) for 96 hr as described [39]. Cells were either not treated or treated with aphidicolin (5 µM) for 24 hrs.

## Supporting Information

Figure S1 TNFRSF6B expression levels are unaffected by *RTEL1^R1264H^*. Whole cell extract (25 µg) prepared from hTERT-immortalized and primary MSK-41 cells were subjected to Western blot analysis using DCR3 (TNFRSF6B) antisera. BJ hTERT and RPE hTERT (an immortalized retinal pigment epithelial cell line) were included as wild type controls. SMC1 serves as a loading control.
(TIF)

Table S1 Exome variant filtering strategy.
(XLSX)

Table S2 Exome coverage statistics.
(XLSX)

Table S3 Variants in telomere- and DDR-related genes and autosomal recessive variants found by whole exome sequencing.
(XLSX)

Table S4 Primers for *RTEL1* locus used in IonTorrent sequencing.
(XLSX)
