# Supplementary figures and images for: A Recessive Founder Mutation in Regulator of Telomere Elongation Helicase 1, RTEL1, Underlies Severe Immunodeficiency and Features of Hoyeraal Hreidarsson Syndrome
Source: PLoS Genet. 2013 Aug 29;9(8):e1003695. doi: 10.1371/journal.pgen.1003695 (PMC3757051; doi:10.1371/journal.pgen.1003695)

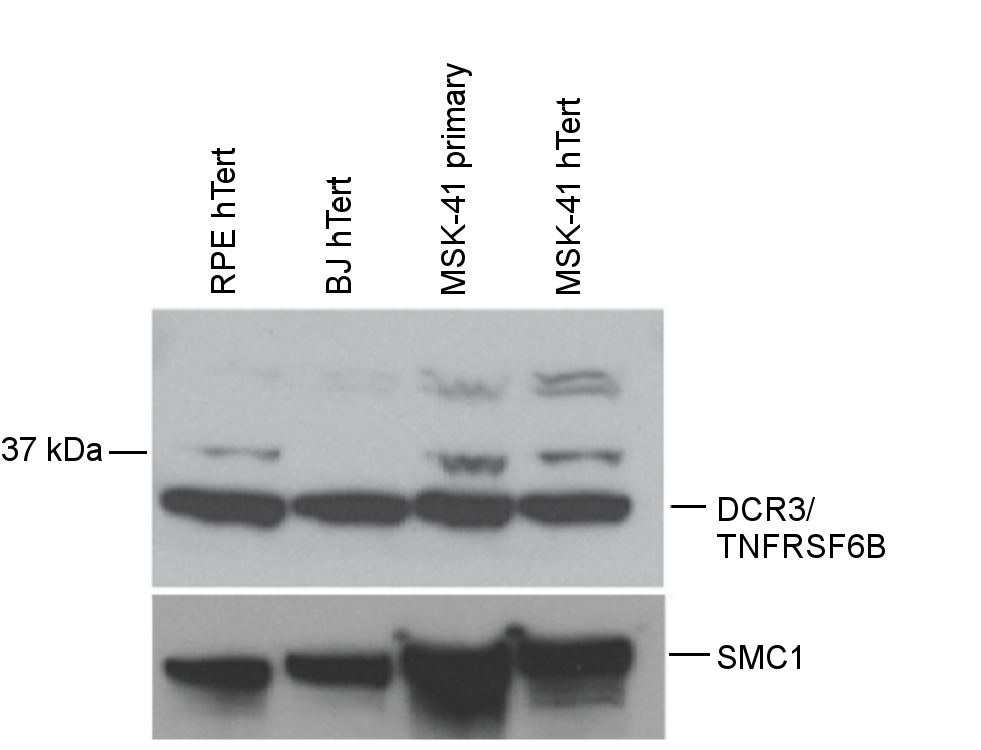

Supplement: Figure S1 — TNFRSF6B expression levels are unaffected by RTEL1R1264H. Whole cell extract (25 µg) prepared from hTERT-immortalized and primary MSK-41 cells were subjected to Western blot analysis using DCR3 (TNFRSF6B) antisera. BJ hTERT and RPE hTERT (an immortalized retinal pigment epithelial cell line) were included as wild type controls. SMC1 serves as a loading control. (TIF) [file pgen.1003695.s001.tif]
